# Effect of Cu-Sn Addition on Corrosion Property of Pressureless Sintered Fe-Cu-Co Substrate Alloys

**DOI:** 10.3390/ma16020728

**Published:** 2023-01-11

**Authors:** Hongliang Tao, Yunzhu Ma, Yuhui Chen, Shuai Du, Haojun Zhou, Yuhang Yin, Yimin Li, Fenghua Luo

**Affiliations:** 1 State Key Laboratory of Powder Metallurgy, Central South University, Changsha 410083, China; 2 Monte–Bianco Diamond Applications Co., Ltd., Foshan 528313, China

**Keywords:** corrosion resistance, pressureless sintering, passivation film, Fe-Cu-Co, Cu-Sn

## Abstract

Fe-Cu-Co prealloyed powder is used for bonding metal of diamond tools. In order to obtain diamond tools with good mechanical properties by pressureless sintering, it is necessary to add Cu-Sn sintering aids. The substrate also has high corrosion resistance requirements, which is conducive to the chemical erosion of diamond tools. This paper mainly studies the effects of Cu-Sn on the corrosion behavior of pressureless sintered Fe-Cu-Co substrate. The results show that the linear contraction rate and relative density of pressureless sintered Fe-Cu-Co alloy at 875 °C reach their peak when the Cu-Sn content is 8 wt.%, 15.13% and 97.5%, respectively. The substrate is mainly composed of α-Fe and Cu-rich phases, and selective corrosion occurs during electrochemical corrosion; namely, α-Fe grains are more prone to corrosion than Cu-rich grains to form porous corrosion surfaces. With the increase in Cu-Sn addition, the volume fraction of the Cu-rich phase increases, the corrosion current density and the passive current density gradually decrease, and the corrosion resistance of the alloy is improved. The amount and integrity of anodic passive film on the Fe-Cu-Co surface increases with the increase in Cu-Sn addition. The oxygen content of the anodic passivation film is lower than that of the active corrosion products of the α-Fe phase, thus reducing the oxygen concentration gradient and slowing down the corrosion. The addition of Cu-Sn is conducive to improving the corrosion resistance of Fe-Cu-Co alloy as the substrate of diamond tools.

## 1. Introduction

There are many kinds of diamond tools, including bonded diamond grits, CVD diamond, loose diamond abrasives, poly crystal diamonds, single crystal diamond, etc. [1,2,3,4]. Among them, the application amount of metal-bonded diamond tools prepared by powder metallurgy is the largest [5]. The methods of preparing metal-bonded diamond tools include hot pressing sintering, hot isostatic pressing, infiltration, and pressureless sintering [6,7,8,9]. A typical hot pressing sintering process is under vacuum, using the following process: linear heating at 5 °C/min up to 700 °C (8 MPa applied pressure), linear heating at 80 °C/min up to 900 °C (35 MPa applied pressure), isothermal step of 5 min (35 MPa applied pressure) [10]. Hot pressing sintering has a low sintering temperature, which can avoid the problem of diamond carbonization due to high-temperature thermal erosion [11,12]. It is also the most widely used method at present. However, the hot pressing sintering pressure is low, the sintering tool density is low, the metal bonding powder is difficult to form metallurgical bonding, and the strength of the substrate is insufficient, which affects the hold ability of the diamond [12]. In addition, this method requires a large number of graphite molds, and the process of powder filling and product removal is cumbersome, which affects production efficiency [13,14]. Hot isostatic pressing can overcome the low density defect of hot pressing sintering, but the cost is high, and there is a risk of thermal erosion of diamond particles caused by overheating [15,16]. No graphite mold is needed for pressureless sintering, and sintering temperature control is relatively easy to achieve [9]. Due to the limitation of carbonization temperature due to thermal erosion of diamonds, the sintering temperature cannot exceed 1000 °C [17]. Therefore, it is difficult to obtain higher density and substrate strength when using commonly used cobalt-based hot pressed sintered metal adhesives in pressureless sintering [9]. In order to improve the strength and diamond-holding force of pressureless sintering diamond tools, the composition of a diamond substrate needs to be designed, such as Fe-Cu-Co [18,19]. Additionally, some sintering aids need to be added [20]. Cu-Sn alloy is a widely used solder material with a low melting point and good metal wettability [21,22], so it is also used as a sintering aid for diamond tools.

Diamond tools are gradually worn down due to wear during use. The wear modes include abrasive wear, adhesive wear, surface fatigue, and chemical erosion wear [17,23,24,25]. Abrasive wear, adhesive wear, and surface fatigue mainly depend on the hardness, strength, and interface conditions of the diamond and bonding metal, which are the issues of greater concern. In practical applications, diamond tools are exposed to high humidity, high acid and alkali, and other harsh environments for a long time, and the substrate is corroded due to environmental corrosion, which reduces the holding force of substrates on diamonds, and affects their service life [23,26,27]. Due to the high chemical resistance of diamond particles and cobalt-based binder materials, which is also an important feature of cobalt-based metals being selected as diamond-bonding metals, chemical erosion wear of hot pressed diamond tools is not a major problem [27,28]. Cu-Sn alloy powder is used as a sintering aid in pressureless sintering [20]. However, it has not been reported whether Cu-Sn affects the corrosion resistance of a diamond substrate, so it is necessary to study the effect of Cu-Sn alloy powder on the chemical corrosion property of cobalt-based bonding metal. In this paper, the prealloyed Fe-Cu-Co powder is used as the matrix powder, and the Cu-Sn alloy powder with different content is added as the sintering aid to prepare the diamond substrate through pressureless sintering. A Tafel polarization curve of the studied alloy was measured using an electrochemical workstation to evaluate the effects of Cu-Sn on the corrosion resistance of Fe-Cu-Co diamond tool substrate materials.

## 2. Material Preparation and Research Methods

Fe-Cu-Co prealloyed powder was prepared by water atomization method. The alloy composition was Cu 28.7 wt.%, Co 12.1 wt.%, and P 0.9 wt.%, with Fe as the balance, wt.% as the mass percentage. The powder size was less than 31 µm. Cu-Sn prealloyed powder was also prepared by water atomization method as a sintering aid. The main component of the alloy was 15.0 wt.% Sn and the balance was Cu. Cu-Sn powder size was less than 38 µm. Five kinds of studied alloys were prepared by adding Cu-Sn powder to Fe-Cu-Co alloy powder. The mixed ingredients and chemical composition of powders are listed in Table 1.

As can be seen from Table 1, with the increase in Cn-Sn addition, the mass ratio of Cu and Sn in the designed alloys increases, while the content of Co, P, and Fe elements slightly decreases. According to the powder mass ratio designed in Table 1, the two kinds of prealloyed powder were weighed and loaded into the vacuum ball mill tank, and evenly mixed for 60 min in the roller mixer. The mixed powder was loaded into the rubber envelope and formed by cold isostatic pressing after vibration. The cold isostatic pressure was 200 MPa and held for 5 min. The sizes of the prepared compactions were 32 mm × 12 mm × 12 mm.

The compactions were placed in a tubular furnace for pressureless sintering, and the sintering atmosphere was an argon-hydrogen mixture with a hydrogen content of 20 vol.%. The heating step during sintering was divided into three stages. First, the room temperature was increased to 200 °C at a rate of 5 °C/min, and the heat was kept for 1 h. Then, the temperature was increased to 600 °C at the rate of 5 °C/min, and the heat was kept for 1 h. Finally, the temperature was increased to 875 °C at a rate of 3 °C/min and kept for 1 h. After sintering, the furnace was cooled to ambient temperature.

The apparent density of the sintered samples was measured by a digital balance (AE124, Shanghai Xunyu, China) with an error within 0.1 mg by the conventional method of weight/volume. The volume of the sample was obtained by the mass reduction caused by the buoyancy of the water. Shrinkage was calculated through the length-direction change of the sintered sample by a digital micrometer with a 0.01 mm resolution. The density and linear shrinkage of the five samples were tested for each alloy, and the average value was taken as the research result. The electrochemical corrosion property of the alloy was tested using an electrochemical workstation (CHI660E, Shanghai Chenhua, China). The corrosion electrolyte used for electrochemical corrosion was 3.5 wt.% NaCl solution. A traditional three-electrode system was adopted. The Pt electrode and saturated calomel electrode (SCE) were used as counter and reference electrodes, respectively. Before each experiment, the samples were cathodically polarized at −1.5 V_SCE_ for 180 s to remove the air-formed oxide, and then at open circuit potential (OCP) in the 3.5 wt.% NaCl solution for 1500–3600 s to form a stable passive film. The test voltage range of potentiodynamic potential was −1.5 V_SCE_ to 0.5 V_SCE_, and the scanning rate was 0.001 V_SCE_/s.

A Shimadzu XRD instrument (D8-ADVANCEX, Brucker, Karlsruhe, Germany) was used to perform phase identification. The X-ray diffraction scanning angle range was selected as 20° to 100° with a 0.02°/s scanning rate and in coupled continuous mode. Scanning electron microscopy (SEM, Quanta FEG 250, FEI, Hillsboro, OR, USA) and scanning electron microscopy (SEM, NOVATM NanoSEM230, FEI, Hillsboro, OR, USA) were used to observe the microstructure of the sintered alloy and the surface morphology of samples before and after electrochemical etching. The selected spot chemical compositions of alloys before and after electrochemical etching were detected by energy dispersive analyzer (EDS).

## 3. Results and Analysis

**Figure 1:**
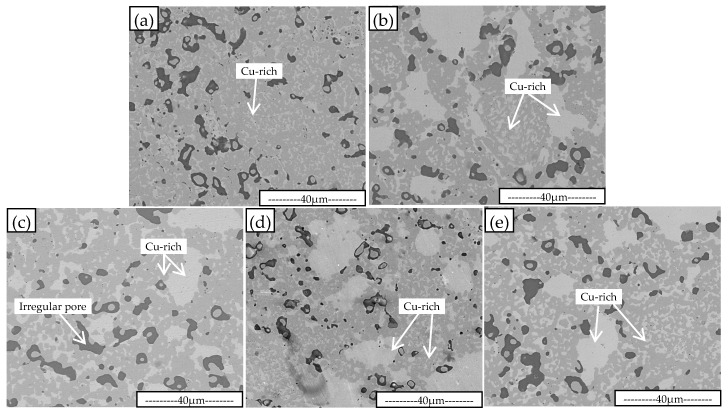
SEM metallographic pictures of sintered Fe-Cu-Co alloys without (**a**) and with 5 wt.% (**b**), 8 wt.% (**c**), 11 wt.% (**d**), 14 wt.% (**e**) Cu-Sn addition.

Figure 1 shows the metallographic microstructure of the five Fe-Cu-Co sintered alloys. As can be seen from Figure 1, there are many pores in the sintered alloys. These pores are irregular in shape and have different sizes.

**Figure 2:**
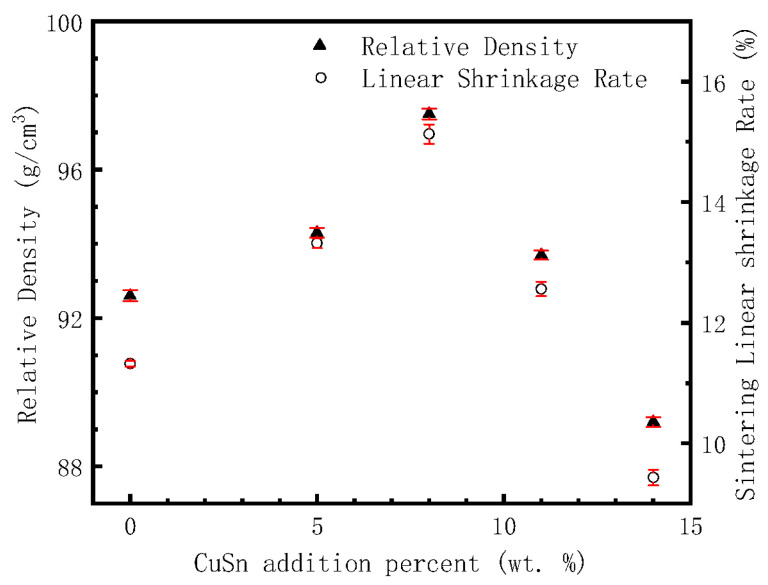
The relations between the relative density and the linear shrinkage rate of sintered alloys to Cu-Sn addition percent.

Figure 2 displays the relationship between the relative density of the sintered alloy and the Cu-Sn addition amount. The relative density is the ratio of the bulk density to the theoretical density of the sintered alloys. The theoretical density is calculated by weighting the alloy composition listed in Table 1. Figure 2 also gives the data for the linear shrinkage, that is, the shrinkage of the compact length after sintering.

It can be seen from Figure 2 that the linear shrinkage rate and the relative density of sintered alloys are synchronous with the change in Cu-Sn addition percent. That is, with the increase in Cu-Sn addition, the linear shrinkage rate and relative density reach peak value when the Cu-Sn content is 8 wt.%, which are 15.13% and 97.5% respectively. It can be seen also that although the sintering temperature is only 875 °C, which is not a very high temperature, the sintering density of the alloy is relatively high. Cu-Sn binary alloy has a peritectic reaction at 799 °C; some liquid phase will be accordingly generated when the content of Sn exceeds 13.5 wt.% [29,30,31]. With the increase in temperature, the amount of liquid phase will be further increased. As the content of Sn in Cu-Sn prealloyed powder is 15 wt.%, the Fe-Cu-Co alloy added with Cu-Sn prealloyed powder will produce a liquid phase during the heating sintering process, forming a liquid phase sintering mechanism [19,20]. In the process of liquid phase sintering, the solid powder particles will rotate and move, realizing the rearrangement of the powder particles, thus increasing the alloy density [32,33]. On the other hand, the existence of a liquid phase can also improve the atomic diffusion ability and promote the sintering process. Table 1 shows that the content of Sn atoms in the studied alloy is not high enough to form a liquid phase relative to the total copper content. As the prealloyed Cu content of Fe-Cu-Co alloy powder is 28.7 wt.%, Sn atoms in the liquid phase will continue to dissolve into the Cu-rich grains during the sintering process, which will lead to the disappearance of the liquid phase and, finally, obtain the complete solid sintered alloy. There is 0.9% P in Fe-Cu-Co prealloyed powder. Due to the low solid solubility of P in Cu, Fe, and other metals, the melting point of P is only 590 °C, so a small amount of P-containing liquid phase can also be formed during sintering [34,35,36]. The high sintering linear shrinkage and relative density comes from the promotion action of liquid phase sintering. However, the sintering process is not only a process of increasing density, but also a process of pore merging and growing. When the volume fraction of the liquid phase is large at the initial stage of sintering, pore formation and growth rate are also fast. The liquid phase should disappear in the later stage, making it difficult to shrink the pore and further improve the density. It can be seen from Figure 1 that the sintering pores have irregular shapes, which are completely different from some round type pores formed in alloys by high temperature solid phase sintering [37,38]. This is because the alloys are mainly composed of α-Fe phase grains, and the high density of powder metallurgical steel should be sintered at a temperature higher than 1120 °C [39,40]. When the liquid phase disappears, the sintering power is greatly weakened, resulting in the difficulty of spheroidizing the residual sintering pores, and the irregular pore can be retained.

**Figure 3:**
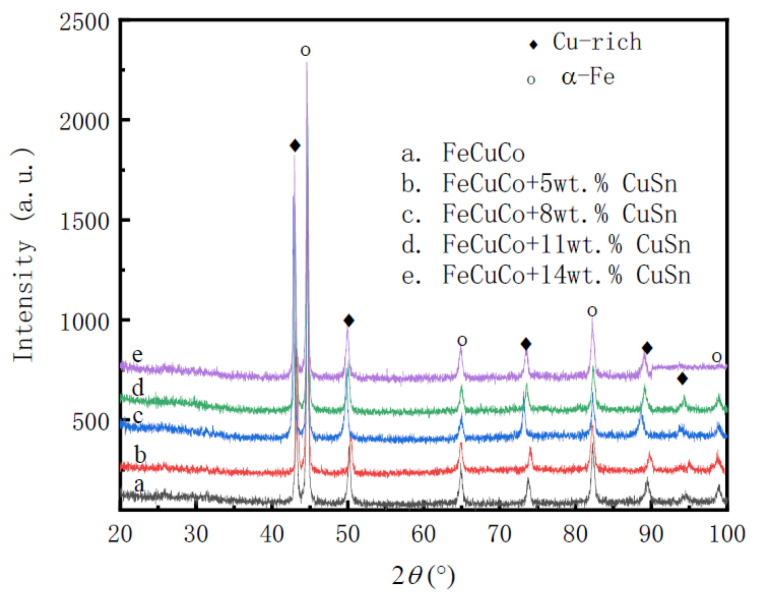
The XRD patterns of sintered FeCuCo alloys.

It can be seen from Figure 1 that the alloys are mainly composed of α-Fe and Cu-rich phases. Generally, the Fe-Cu-Co alloys used as diamond substrates are composed of these two phases [26,27]. As a Sn atom has large solid solubility in the Cu phase at elevated temperatures, it will not form a new phase alone. It can be seen from Figure 1 that there are large-sized Cu-rich phase aggregate grains in the alloys with Cu-Sn added. The grains of Cu-rich phase in alloy with 8 wt.% Cu-Sn addition are slightly coarser than in other alloys, indicating the process of Cu atom aggregation and diffusion is more complete, thus obtaining higher density. Figure 3 shows the XRD diffraction patterns of studied alloys, which indicates that the phase composition of the studied alloy is mainly α-Fe and Cu-rich phase.

**Figure 4:**
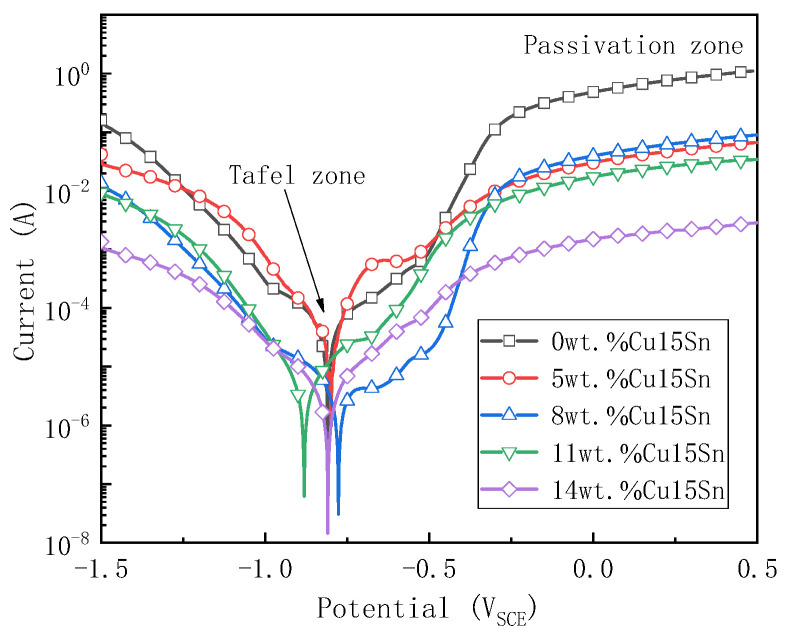
Effects of Cu-Sn addition on potentiodynamic polarization curves of Fe-Cu-Co pressureless sintering alloys.

Figure 4 shows the potentiodynamic polarization curves of the studied Fe-Cu-Co alloys. It can be seen from Figure 4 that the five kinds of pressureless sintered Fe-Cu-Co alloys have passivation zones at potentials above −0.2 V_SCE_. With the increase in Cu-Sn addition, the current density in the passivation zone gradually decreases. Specifically, when the voltage is 0 V_SCE_, the amount of Cu-Sn added is 0, 5 wt.%, 8 wt.%, 11 wt.%, and 14 wt.%, respectively, and the current density corresponding to each alloy is 0.482 A/cm^2^, 0.030 A/cm^2^, 0.039 A/cm^2^, 0.017 A/cm^2^, and 0.0015 A/cm^2^, respectively. It can be seen that the addition of Cu-Sn affects the formation and dissolution of the passive film during the electrochemical corrosion of the alloy. With the increase of Cu-Sn addition, the current density in the passivation zone decreases, indicating that the dissolution rate of the passivation film slows down.

**Figure 5:**
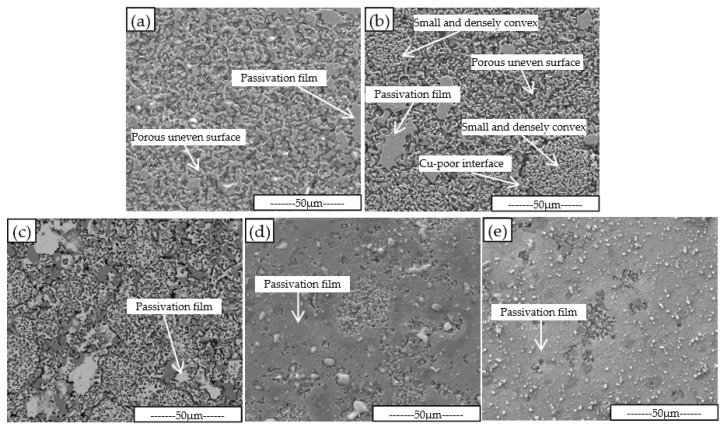
SEM surface morphology of alloys with (**a**) 0%, (**b**) 5%, (**c**) 8%, (**d**) 11%, and (**e**) 14% Cu-Sn addition after electrochemical test.

In order to compare the difference of electrochemical corrosion resistance properties of different alloys, the analysis software attached to the electrochemical workstation was used to obtain the corrosion current density I_corr_ and corrosion potential E_corr_ by Tafel region extrapolation method. Table 2 lists the corrosion potential and corrosion current density of the five alloys. It can be seen from Table 2 that, with the increase in Cu-Sn addition percent, the corrosion current density of the alloy slowly decreases. When the addition of Cu-Sn increases to 11 wt.%, the corrosion current density of the alloy shows an order of magnitude different to that of the alloy without Cu-Sn addition. When the addition of Cu-Sn is 14 wt.%, the corrosion current density is only 4.30 × 10^−6^ A/cm^2^. According to Faraday’s law, the higher corrosion current density of an alloy means a faster corrosion rate, and worse corrosion resistance [38,41]. With the increase in Cu-Sn content, the corrosion current density of the alloy decreases, indicating that the addition of Cu-Sn to Fe-Cu-Co can improve the corrosion resistance of the substrate. Figure 5 shows the SEM pictures of the surface morphology of the alloy after the electrochemical polarization curve test. The sample surfaces were cleaned with anhydrous ethanol before observing the surface morphology.

It can be seen from Figure 5 that there is only a small amount of residual anodic passivation film on the surface of the alloy without Cu-Sn. With the increase in Cu-Sn addition, the area of passivation film on the alloy surface obviously increases. The passivation film on the surface of the Fe-Cu-Co alloy added with 11 wt.% and 14 wt.% Cu-Sn takes up most of the surface area. The surface area not covered by the passivation film shows obvious uneven corrosion. Some grains have a fast corrosion rate, while others have a slow corrosion rate, resulting in porous uneven surfaces. Figure 3 shows that Fe-Cu-Co alloy is mainly composed of α-Fe and Cu-rich phases. Because the potential of the Cu-rich phase is higher than the α-Fe phase, this will cause selective corrosion of α-Fe phase grains during electrolytic corrosion and form the convex feature of Cu-rich phase grains on the corrosion surface. The convex area and size in Figure 5a are equivalent to those of the Cu-rich phase grains shown in Figure 1a, which also indicates the selective corrosion of α-Fe grains. As shown in Figure 1, after adding Cu-Sn, a large-sized Cu-rich agglomeration area will appear, but this feature is not obvious in Figure 5. Through analysis of Figure 5b, it can be seen that there are smaller and densely convex areas, and there are obvious continuous and deeper corrosion boundaries between this area and other parts. At 875 °C, the maximum solid solubility of Sn in Cu exceeds 8.0 wt.%, while the solid solubility of Sn atom in α-Fe is very limited. Therefore, a Sn atom and Cu phase form a uniform solid solution at sintering temperature. However, the solid solubility of Sn in the Cu phase is very limited at room temperature. The Cu-Sn solid solution will form a α-Cu and ε-Cu_3_Sn eutectoid structure at room temperature [28,29,30]. Therefore, the large-sized Cu-rich phase grain seen in Figure 1 is actually a α-Cu and ε-Cu_3_Sn eutectoid structure. Selective corrosion will also occur during electrolytic corrosion, forming the small and densely convex zone marked out in Figure 5b. Since the sintering process is an instantaneous liquid phase process, when these large-sized Cu-rich phase grains are formed, the Cu atoms of the surrounding area preferentially diffuse to these Cu-rich phase grains, resulting in the formation of a Cu-poor interface, so an obvious corrosion boundary is formed around them.

**Figure 6:**
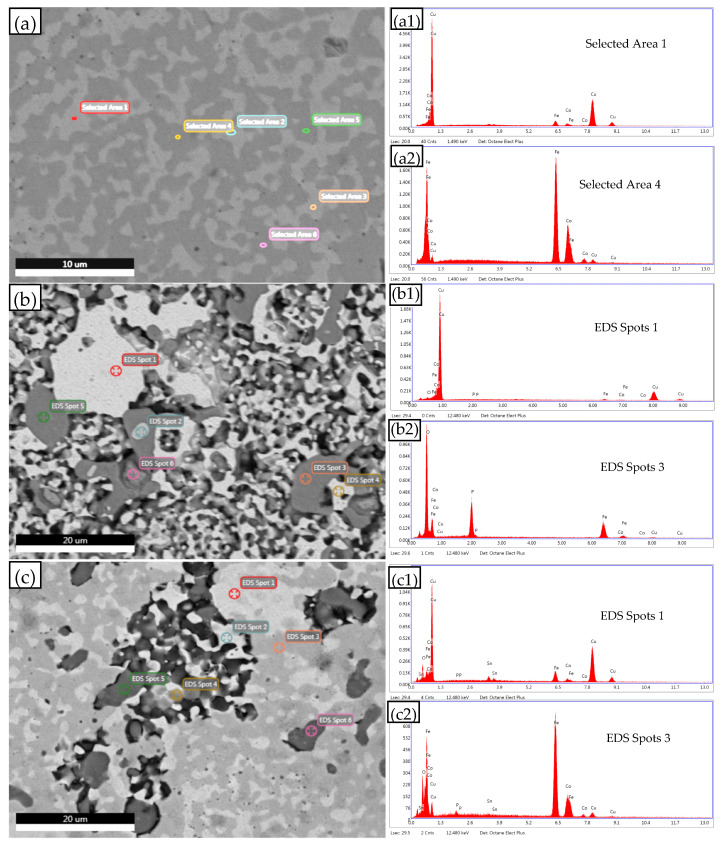
EDS analysis with SEM of Fe-Co-Cu alloy (**a**) before electrochemical test without Cu-Sn addition, (**b**) after electrochemical test without Cu-Sn addition, and (**c**) after electrochemical test with 11 wt.% Cu-Sn addition. X-ray EDS spectrum of selected area (**a1**) 1 and (**a2**) 4 in (**a**), spots (**b1**) 1 and (**b2**) 3 in (**b**), spots (**c1**) 1 and (**c2**) 3 in (**c**).

The chemical composition of the selected area was analyzed using the EDS instrument attached to the electronic scanning microscope. Figure 6 shows the backscatter photos and EDS spectra of the selected areas or spots. The chemical composition analysis results of EDS are listed in Table 3. Figure 6a shows the BSE photos of Fe-Cu-Co alloy without Cu-Sn alloy powder before electrochemical corrosion. Six areas were selected from Figure 6a for EDS composition analysis, corresponding to α-Fe and Cu-rich phases that have three selected areas, respectively. It can be seen from the results in Table 3 that there are about 90% Cu atoms in the Cu-rich phase, and there is a high content of Fe element, while the content of Co element is low. At a high temperature, the solid solubility of Fe in Cu is higher than that of Co [42,43,44], which will be retained after sintering. It can also be seen from Table 3 that the Co element mainly appears in the α-Fe phase. Element P is not found in either phases of Figure 6a, because the solid solubility of element P in α-Fe and Cu-rich phases is very limited, so they tend to be enriched at defects, such as grain boundaries and sintering pores [45].

Figure 6b shows the BSE picture of Fe-Cu-Co alloy without Cu-Sn alloy powder after electrochemical testing. The results in Table 3 show that spots 1 and 4 in Figure 6b are Cu-rich phases. Compared with before the electrochemical test, Fe and Co elements decrease, Cu elements increase, and a small amount of P and O elements appear. This shows that the purity of the Cu-rich phase is improved under selective corrosion. At the same time, the P element in the alloy forms some oxides and deposits on the corrosion surface. The contents of Fe, P, and O elements in spots 3 and 5 in Figure 6b are very high, but the contents of Cu and Co are low. It can be seen that these areas are mainly composed of oxides of Fe and P, indicating the surface corrosion products of α-Fe phase. It also shows that the Co element appears less in these oxides. In order to distinguish its characteristics, it is indicated in Table 3 as “α-Fe active corrosion products”. The area corresponding to spot 2 in Figure 6b has high Fe, Co, and P contents, but low oxygen content. It is indicated that this area is a naked α-Fe phase area, indicated in Table 3 as “α-Fe clean”. The area corresponding to spot 6 in Figure 6b contains a high content of Cu and high contents of Fe, P, and O, which is obviously composed of a mixed Cu-rich phase and α-Fe active corrosion products. The EDS composition analysis results of each area in Figure 6b show that Cu-rich is not prone to corrosion, while the α-Fe phase preferentially corrodes to form a large number of oxides. This result further indicates that selective corrosion will occur on the exposed surface of the Fe-Cu-Co alloy.

Figure 6c shows BSE photos of the Fe-Cu-Co alloy with 11 wt.% of Cu-Sn addition after electrochemical testing. EDS analysis mainly reflects the chemical composition of the passivation film on the corroded surface. It can be seen from Table 3 that spot 1 in Figure 6c is obviously a Cu-rich phase corrosion surface passivation film. In addition to the main element Cu, the Sn element content in this spot is high, and there are P and O elements at the same time. The corresponding spots 3, 4, and 5 are obviously passivation films of the α-Fe phase. In addition to the main element Fe, the content of Co element in these passivation films is high, and there are also P and O elements. Although the passivation film in Figure 6c also contains more O elements, compared with spot 3 and 5 in Figure 6b, their oxygen content significantly decreases. The decrease of oxygen content in the surface passivation film would reduce the oxygen concentration gradient between the surface and the alloy interior, which is conducive to slowing down the corrosion process. The results in Figure 4 and Table 2 show that with the increase in Cu-Sn addition, thecorrosion current density and the passive current density of the alloy decrease. On the one hand, the increase of Cu phase content in the alloy reduces the corrosion rate of the alloy, and on the other hand, the area of the anodic passivation film on the alloy surface increases, which improves the integrity of the passivation film, thus reducing the corrosion rate of the passivation zone. It can be seen that the addition of Cu-Sn is conducive to improving the corrosion resistance of Fe-Cu-Co alloy as the substrate of diamond tools.

## 4. Conclusions

(1) The linear yield and relative density of the pressureless sintered Fe-Cu-Co alloy at 875 °C reached their peak when the Cu-Sn content was 8 wt.%, which were 15.13% and 97.5%, respectively. However, with the increase in Cu-Sn addition, the corrosion current density and the passive current density gradually decreased, and the corrosion resistance gradually improved. The addition of Cu-Sn was conducive to improving the corrosion resistance of the Fe-Cu-Co alloy as the substrate of diamond tools.

(2) Fe-Cu-Co alloy mainly consisted of α-Fe and Cu-rich phases, and selective corrosion occurred during electrochemical testing; α-Fe grains were preferred to Cu-rich grains to form porous corrosion surfaces. With the increase in Cu-Sn addition, the volume fraction of Cu-rich phase increased and the corrosion resistance of the alloy increased.

(3) With the increase in Cu-Sn addition, the integrity of anodic passive film on Fe-Cu-Co surface was improved. The oxygen content ratio in the anodic passivation film of the α-Fe phase was lower than that of active corrosion products, which reduced the oxygen concentration gradient and slowed down corrosion.

## Figures and Tables

**Table 1:** Powder mixed ingredients and chemical composition of alloys.

Alloys	Powder Mixed Ingredients, wt.%		Chemical Composition, wt.%				
	Fe-Cu-Co	Cn-Sn	Cu	Co	Sn	P	Fe
1	100	0	28.70	12.10		0.90	58.30
2	95	5	31.52	11.50	0.75	0.86	55.39
3	92	8	33.20	11.13	1.20	0.83	53.64
4	89	11	34.89	10.77	1.65	0.80	51.89
5	86	14	36.58	10.41	2.10	0.77	50.14

**Table 2:** Effect of Cu-Sn addition on corrosion potential and current of Fe-Cu-Co alloys.

Cu-Sn Addition Percent (wt.%)	E_corr_ (V_SCE_)	I_corr_ (10^−6^A/cm^2^)
0	−0.81	78.53
5	−0.80	38.16
8	−0.78	11.43
11	−0.88	5.16
14	−0.81	4.30

**Table 3:** EDS selected zone chemical composition analysis results of Figure 6.

Alloy and State	Selected Area or Spots	Chemical Compisition (wt.%)						Phase
		Fe	Cu	Co	Sn	P	O	
Fe-Cu-Co alloy without Cu-Sn addition before electrochemical test, EDS area refer to Figure 6a	1	7.04	89.94	3.01				Cu-rich
	2	6.86	90.01	3.12				Cu-rich
	3	6.08	91.11	2.81				Cu-rich
	4	68.29	3.43	28.36				α-Fe
	5	68.31	4.01	27.68				α-Fe
	6	68.26	5.16	26.57				α-Fe
Fe-Cu-Co alloy without Cu-Sn addition after electrochemical test, EDS spots refer to Figure 6b	1	3.96	92.57	1.88		0.34	1.25	Cu-rich
	4	4.41	92.03	1.44		0.47	1.66	Cu-rich
	3	22.84	3.00	0.89		25.61	47.66	α-Fe active corrosion products
	5	48.91	2.03	0.63		17.74	30.70	α-Fe active corrosion products
	2	48.9	3.55	23.95		21.62	1.97	α-Fe clean
	6	28.03	37.17	5.38		9.71	19.70	Cu-rich + α-Fe active corrosion products
Fe-Cu-Co alloy with 11 wt.% Cu-Sn addition after electrochemical test, EDS spots refer to Figure 6c	1	11.77	74.34	3.31	5.66	0.39	4.52	Cu-rich passivation film
	2	32.70	44.73	8.41	4.19	1.81	8.17	Cu-rich + α-Fe passivation film
	4	50.80	32.10	11.25	0.79	0.42	4.63	α-Fe + Cu-richpassivation film
	3	72.35	4.92	16.04	0.96	0.59	5.14	α-Fe passivation film
	5	72.42	3.30	16.86	0.46	1.41	5.55	α-Fe passivation film
	6	69.33	6.79	16.74	0.46	0.22	6.46	α-Fe passivation film

## Data Availability

Not applicable.
